# Low-Cost and Highly Sensitive Wearable Sensor Based on Napkin for Health Monitoring

**DOI:** 10.3390/s19153427

**Published:** 2019-08-05

**Authors:** Liping Xie, Peng Chen, Shuo Chen, Kun Yu, Hongbin Sun

**Affiliations:** 1 College of Medicine and Biological Information Engineering, Engineering Research Center of Medical Imaging and Intelligent Analysis, Ministry of Education, Northeastern University, Shenyang 110169, China; 2 School of Chemical and Biomedical Engineering, Innovative Centre for Flexible Devices, Nanyang Technological University, Singapore 637459, Singapore; 3 Department of Chemistry, Northeastern University, Shenyang 110819, China

**Keywords:** napkin tissue, wearable, highly sensitive, capacitive sensor, wrist pulse

## Abstract

The development of sensors with high sensitivity, good flexibility, low cost, and capability of detecting multiple inputs is of great significance for wearable electronics. Herein, we report a napkin-based wearable capacitive sensor fabricated by a novel, low-cost, and facile strategy. The capacitive sensor is composed of two pieces of electrode plates manufactured by spontaneous assembly of silver nanowires (NWs) on a polydimethylsiloxane (PDMS)-patterned napkin. The sensor possesses high sensitivity (>7.492 kPa^−1^), low cost, and capability for simultaneous detection of multiple signals. We demonstrate that the capacitive sensor can be applied to identify a variety of human physiological signals, including finger motions, eye blinking, and minute wrist pulse. More interestingly, the capacitive sensor comfortably attached to the temple can simultaneously monitor eye blinking and blood pulse. The demonstrated sensor shows great prospects in the applications of human–machine interface, prosthetics, home-based healthcare, and flexible touch panels.

## 1. Introduction

Wearable sensors have gained considerable attention in academics and industry because of their promising applications in personal healthcare [1], human-activity monitoring [2], and artificial intelligent robotics [3,4,5]. Due to their low modulus, light weight, flexibility, high sensitivity, and stretchability, wearable sensors are conformally in contact with the surface of organs or skin, which paves a new way for fitness tracking, disease diagnosis, and risk prevention by monitoring of human motions ranging from tiny physiological signals (e.g., pulse, heart rate, respiration, and phonation) to substantial movements (e.g., muscle movement) [6,7,8,9,10,11]. Different strategies have been introduced based on various detection mechanisms, such as piezoelectric, resistive, and capacitive principles, with multiscale architectures [12,13,14,15,16,17]. However, most of the methods rely on microelectronics fabrication processes and involve complex fabrication processes, such as ultraviolet exposure, oxygen-plasma, vacuum deposition of films, spin-coating, photolithography, wet and dry etching, and transfer-printing. Despite them having been proved to be effective, the high cost associated with expensive instruments and materials limits their applications. Some low-cost and highly sensitive flexible sensors were developed, however, the fabrications involved multiple steps [18,19]. It is still a challenge to develop a flexible sensor with high sensitivity in a cost-effective way through a facile manufacturing process.

Paper has been widely used as a support material for fabrication of sensors and devices in analytical and clinical analysis due to its portability, low cost, ready availability, light weight, and eco-friendliness [20,21,22,23,24]. Fluids can transport through hydrophilic cellulose by wicking mechanism without the aid of an external force. The natural unique hierarchical and mesoporous structures of paper enable plenty of new applications beyond their traditional use. Paper-based wearable sensors open a new door for the fabrication of wearable sensors [25]. Due to their ease of availability, renewability, and mechanical flexibility, paper-based electronic devices have drawn a wide range of attention [26,27,28,29]. Up to now, a stable transparent conducting electrode with exceptional and stable optoelectronic properties was fabricated by embedding Ag nanowires (NWs) into the surface of transparent paper [30]. Graphene was transferred on a super-clear paper to fabricate a multitouch touch screen [31]. Paper-based triboelectric nanogenerators with rationally designed interlocking kirigami structures were fabricated [32]. Paper-based piezoresistive sensor generated by conductive materials patterned on a paper substrate could measure forces down to 120 mN [33]. However, none of these paper-based electronic devices are used to monitor physical signals (e.g., wrist pulse). Recently, Tao and his colleagues provided a graphene-paper pressure sensor with excellent performance in the range of 0−20 kPa [34]. However, it needed a high temperature of 250 °C and a long time (5 h) to prepare the sensor. Moreover, although they demonstrated the sensor possessed an ultrahigh sensitivity of 17.2 kPa^−1^, the sensor didn’t distinctly discriminate the characteristics of wrist pulse and the testing curve was not a standard wrist pulse wave. Most of the previously reported paper sensors are fabricated through expensive and complex processes without controllable sensitivity for measuring wrist pulse.

Here, a novel, facile, and low-cost method for fabrication of sensitive napkin-based sensor was developed without any instruments and high skills. We fabricated a napkin-based capacitive sensor by automatically immobilizing silver nanowires (NWs) on a hydrophilic/hydrophobic-patterned napkin using a folded paper coated with cured polydimethylsiloxane (PDMS). The napkin-based sensor was applied to many practical recognition applications, such as sensing human body motions (finger motion, eye blinking) and even distinct discrimination of tiny wrist pulse.

## 2. Experimental Materials and Experimental Methods

### 2.1. Reagents and Instruments

Polydimethylsiloxane (PDMS) elastomer kit was bought from Dow Corning (Midland, MI, USA). Medical polyurethane (PU) films were purchased from Jiaxing Meson Medical Materials Co. Ltd. (Zhejiang, China). Silver NWs in ethanol solution (10 mg mL^−1^) were brought from Shanghai Bohan Chemical Technology Co. Ltd. (Shanghai, China). Scanning electron microscopy (SEM) imaging was carried out with a JSM-7900F field emission scanning electron microscope operating at an acceleration voltage of 10 kV (JEOL, Tokyo, Japan). The capacitances of the flexible sensor were measured with a precision LCR meter E4980A (Keysight, CA, USA). Pressure performance of the sensors was recorded by a digital tensile testing machine bought from Yueqing Handpi instrument co. LTD (Hunan, China).

### 2.2. Experimental Methods

A folded paper was obtained by cutting along a digitally designed pattern on a common printing paper. Uncured PDMS made of PDMS pre-polymer and curing agent in a mass ratio of 10:1 was homogeneously applied to the folded paper, then, a piece of napkin was inserted into the folded paper. PDMS from the folded paper infiltrated into the napkin simultaneously from the front and back sides. The uncured PDMS-modified napkin was heated on a hot plate at 60 °C for 3 min. The hydrophobic agent (PDMS) in the napkin was cured to form a hydrophilic/hydrophobic pattern on the napkin, then, several drops of silver NWs in ethanol solution were added to the hydrophilic area of the napkin. The silver NWs solution was driven by the wicking action of the napkin and formed a conductive network on the surface of the napkin. After the evaporation of the ethanol, a PU film was used to seal the conductive network to form an electrode plate. Two pieces of electrode plates coupled to construct a capacitive sensor.

## 3. Results and Discussion

### 3.1. Fabrication of Napkin-Based Wearable Sensor

**Figure 1:**
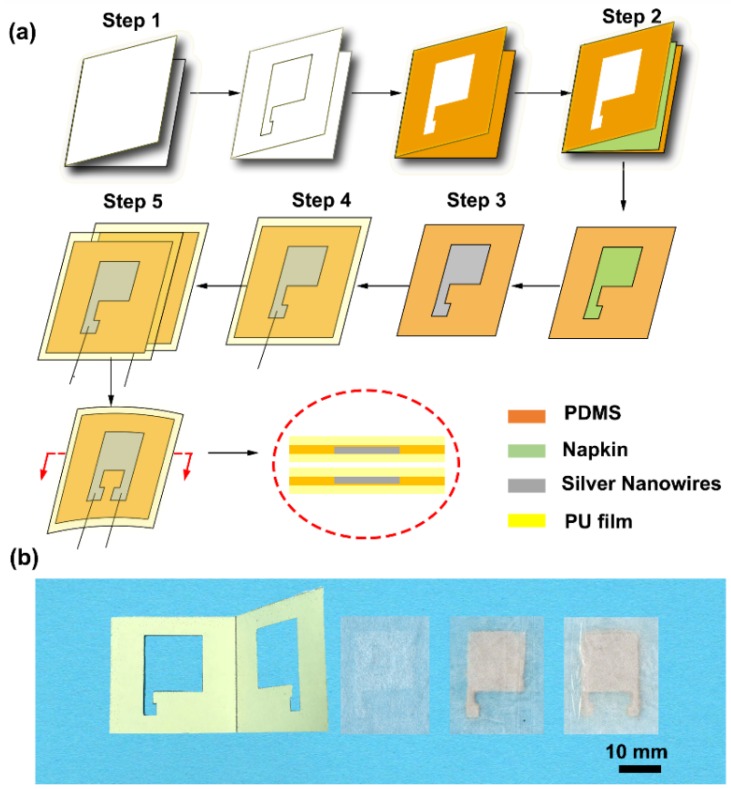
Schematic illustration of the fabrication of the napkin-based capacitive sensor. (**a**) Fabrication process. Step 1: A folded paper with hollowed pattern coated with polydimethylsiloxane (PDMS). Step 2: A piece of napkin is inserted into the folded paper, resulting in hydrophilic and hydrophobic pattern on the napkin. Step 3: Adding silver nanowires (NWs) in ethanol solution to the patterned napkin. Step 4: Packing it with PU film. Step 5: Two pieces of the electrode plates are coupling to form a capacitive sensor. (**b**) Photographs of the folded paper, hydrophilic–hydrophobic patterned napkin, single-electrode plate, and capacitive sensor.

A napkin-based wearable sensor was achieved by a simple folded paper-based method shown in the schematics (Figure 1a). The patterned folded paper was fabricated by cutting along the designed pattern using a knife (Figure 1b). Uncured PDMS was applied to coat the surface of the folded paper. A layer of napkin with a weight of 0.116 g was inserted into the modified folded paper and formed a sandwich structure so that the uncured PDMS infiltrated into the napkin. The designed pattern was entirely formed on the napkin, and the total weight of the PDMS-modified napkin changed to 0.180 g. The specific region of the napkin was hydrophobized by the cured PDMS due to its natural hydrophobicity. The silver NWs in ethanol solution were added to the hydrophilic region consisting of porous cellulose. The napkin allowed automatic loading and patterning of silver liquid driven by capillarity action. Silver conductive network was formed on the surface of the napkin after the evaporation of the ethanol. A piece of medical PU film was brought into conformal contact with the napkin with a silver conductive pattern to form a single conductive electrode plate with a weight of 0.248 g. Two pieces of conductive electrode plates were brought together to construct a capacitive sensor (Figure 1b).

### 3.2. Optimization and Characterization of the Napkin-Based Sensor

**Figure 2:**
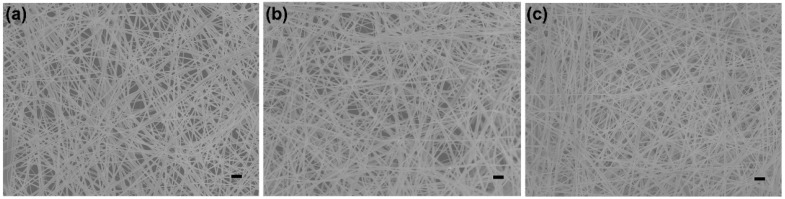
Optimization of the areal density of the silver NWs on a napkin. SEM images of the silver NWs on napkins with the density of 0.67 mg cm^−2^ (**a**), 1 mg cm^−2^ (**b**), and 1.33 mg cm^−2^ (**c**), respectively. The scale bars are 1 μm.

**Figure 3:**
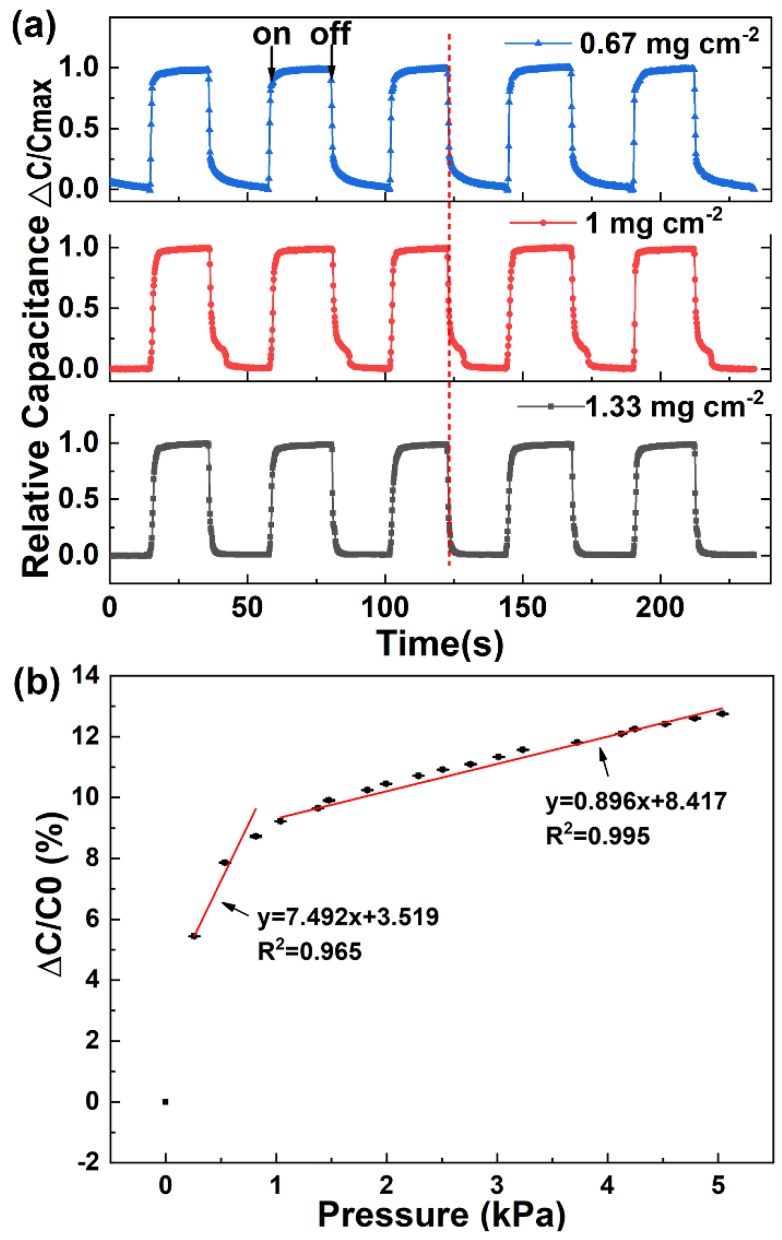
Optimization and characterization of the electrical properties and performances of the capacitive sensor. (**a**) Changes in capacitance under pressure on and off. (**b**) Relative change in capacitance with applied pressure. Error bars show standard deviation with N = 3.

In order to obtain a capacitive sensor with excellent electronic response, we optimized the areal density of silver NWs on the napkin. Silver NWs with diameters of ~200 nm are staggered together and form a conductive network connection (Figure 2). Thus when the areal density of silver NWs on the napkin increases, the conductive network is enhanced. The sheet resistance of the conductive network decreases with the increase of the silver NWs areal density on the napkin (Figure S1). When the density of the silver NWs reaches 1.33 mg cm^−2^, the silver NWs nearly cover the whole area of the napkin (Figure 2c), and the sheet resistance is as low as 0.42 Ω sq^−1^ with good electrical conductivity. The low sheet resistance of the conductive network is attributed to the high density of the silver NWs and the long silver NWs (~100 µm) being able to form a more effective conductive network. In addition, the relative capacitances of the napkin with different densities of silver NWs under the applied pressure level of 6.5 kPa were compared. As shown in Figure 3a, all the sensors with different densities of silver NWs exhibit good pressure-responsive properties. The change of electronic capacitance keeps pace with the variation of repeatedly-applied pressure. When the pressure is on, the capacitance of the sensors increases due to the decrease in the average distance between the two electrode plates. When the pressure is off, the capacitance immediately deduces to its original state. However, as shown in the highlighted part by the dashed line, the transient pressure response of the capacitive sensor is improved as the increase of the silver NWs density. When the density of the silver NWs reaches 1.33 mg cm^−2^, the response curve of the capacitance under the pressure of the 6.5 kPa is close to rectangular pulses. Thus, 1.33 mg cm^−2^ of silver NWs was selected as the suitable areal density for fabrication of the napkin-based sensor. We further studied the resolution of the conductive pattern produced by this method. In Figure S1, a PDMS-modified napkin was achieved with different widths of rectangles by the folded paper. Silver NWs was applied to the hydrophilic region to highlight the channel regions. The minimal width of 100 μm was achieved by the method (Figure S2). Limited by the wide diameter of pipette tips, the pattern of the corresponding silver NWs line is a little blur. The resolution relies on the lateral spreading of the blocking agent PDMS. Compared with the previously reported hydrophilic and hydrophobic PDMS-based patterns, the resolution achieved by the folded paper method is much higher [35,36]. There are several reasons attributed to the high resolution. Firstly, the folded paper improves the PDMS penetration rate and reduces the lateral spreading of the PDMS. Secondly, the napkin is as thin as 50 μm, which significantly decreases the diffusion distance and reduces the chance of lateral spreading of PDMS.

To evaluate the electronic performance of the as-fabricated sensor, the pressure response of the capacitive sensor was measured and calculated. The measurement setup was composed of a motorized vertical stage, a force gauge, and an LCR meter, which was applied to register the capacitances of the sensor under different pressures. The pressure sensitivity (S) of the capacitive sensor is expressed as Equation (1):

(1) $$S=(\Delta C/C_{0})/\Delta P,$$

where *C* and *C*_0_ refer to capacitances with and without the pressure, ΔP represents the change of the applied pressure [37]. According to the definition, pressure sensitivity S expressed as the slope of the traces is usually applied to evaluate the performance of a capacitive sensor [38]. By analyzing the relative capacitances in corresponding with the applied pressure, a bilinear relationship between the relative capacitance and the applied pressure was obtained (Figure 3b). The pressure sensitivity, averaged over 3 sensors, is 7.492 kPa^−1^ in low-pressure regime (*P* < 1 kPa) and 0.896 kPa^−1^ at higher pressures (1 < *P* < 5 kPa). The high sensitivity in the low-pressure regime enables the sensor to distinguish tiny pressure signals, especially, weak physiological signal (e.g., wrist pulse, respiration). The relatively lower sensitivity at higher pressures extends the application of the sensor. It can be applied to detect gentle touch and figure motion. The high performance of the napkin-based sensor is sufficient for detection of physical signals.

### 3.3. Monitoring Finger Motion and Eye Blinking

**Figure 4:**
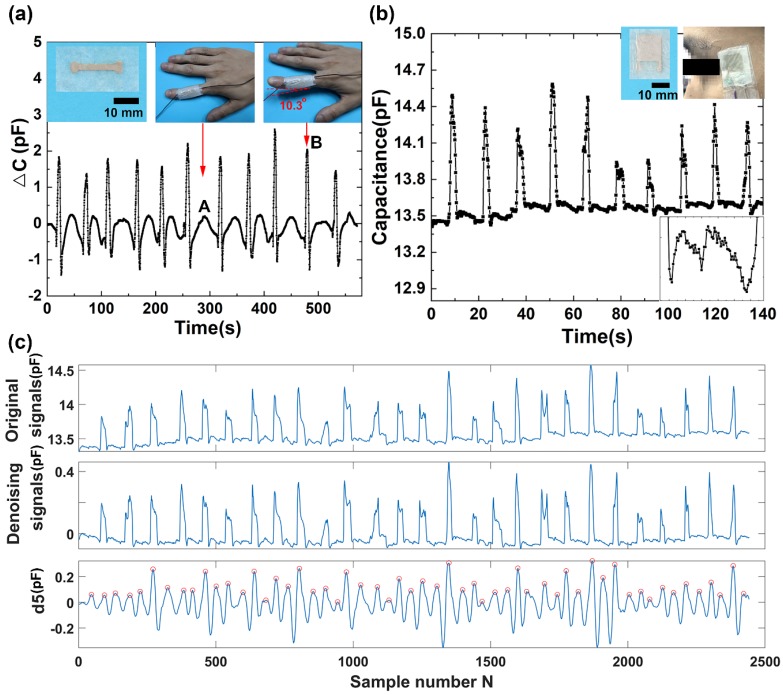
Capacitive sensor for detection of finger motion and eye blinking. (**a**) Capacitance profile of finger motion. (**b**) Capacitance profile of eye blinking. (**c**) The 5th level wavelet high-frequency coefficient decomposed from the original eye blinking signal (d5).

Physical movement measurement is of great importance for healthcare and robotics. The improvement of the upper paralytic limb function for stroke survivors mostly depends on the functional control of their hands [39]. The stroke survivors need to take a lot of hand training before they use the limb in daily activities. Previous studies have shown that the repetitive finger training by flexion and extension movements is effective in improvement of hand function after stroke [40]. To illustrate the application of the capacitive sensor for large pressure sensing, we placed the sensor on the proximal phalanx of a volunteer’s index finger. As shown in Figure 4a, at the initial state, the finger is straight where there is nearly no electrical capacitance change (state A). When the finger lifts up about 10.3° from the horizontal plane, an increase in the capacitance is observed (state B). When the finger restores to the straight state, the capacitance resumes state A. Similar pressure response is achieved by repeating the finger motion in the same manner. The results show that the capacitive sensor can capture the finger motions effectively. The capacitance of the sensor can reflect the movement of the finger from lifting up to putting down. The sensitively tracking of finger motion provides a way for stroke patients’ hand rehabilitation.

In addition, spontaneous eye blinking is considered to be a suitable indicator for fatigue diagnostics during many tasks of human being activity, and the measurement of an eye blink parameter provides reliable information for eye-controlled systems from a human–machine interface. The system may be used for detecting basic commands to control some instruments, robots, or any other applications by people with limited upper-body mobility. We applied the as-fabricated sensor to detect eye blinking. The sensor was attached to the temple of a volunteer by a PU tape (Figure 4b). A precision LCR meter connected to the sensor by wires was used to record the capacitance of the sensor. Eye blinking caused the muscles’ movements around the eye, which changed the distance between the electrode plates. Significant increases of the capacitances were obtained during eye blinking. The repeated eye blinking produced a similar capacitive response. The capacitance peak values of the sensor responding to eye blinking are a little different due to the various intensities of eye blinking. During the unblinking process, the capacitances are at the baseline state. More interestingly, there seems a regular signal in the baseline. We further enlarge one part of the baseline, and a regular signal is shown in the enlarged image in Figure 4b. To further investigate the signal, we take advantage of wavelet transform to analyze the recorded signals. Wavelet technique has been widely employed to analyze various physiological signals [41]. The method is preferred over signal frequency domain filtering because it can maintain signal characteristics even while reducing noise [42]. Wavelet transform employs extensive time windows for low frequencies; and short time windows for higher frequencies, resulting in good time-frequency analysis [43]. The output of the first high pass and low pass filters are referred to as the approximation and detailed coefficients, represented by a_1_ and d_1_, respectively. The a_1_ is further disintegrated, and the procedure is repeated until the specified number of decomposition levels is reached. In this experiment, wavelet functions Daubechies wavelet db8 is used for the wavelet transform. After wavelet decomposition of 8 levels, the original signal is decomposed into low-frequency approximation coefficients a_1_–a_8_ (Figure S3) and high-frequency detail coefficients d_1_–d_8_ (Figure S4). The noisy signal corresponding to the relevant part of the low-frequency detail a_8_ is removed from the recorded eye blinking signals, and the remaining parts are reconstructed to obtain the denoising signal (Figure 4c). The sequence of the eye blinking wave is a long repeatedly blinking wave. The high-frequency detail coefficients at level 5 are isolated and shown in Figure 4c. By calculating the characteristic peaks marked with circles in d_5_, we can obtain that the frequency of the signal is 70 times per minute. The analytical result coincides well with the pulse rate of a healthy person, whose pulse rate locates in the range from 60 to 100 times per minute.

All these results show that the periodic signal observed during the eye blink is properly the blood pulse wave.

### 3.4. Detection of Wrist Pulse

**Figure 5:**
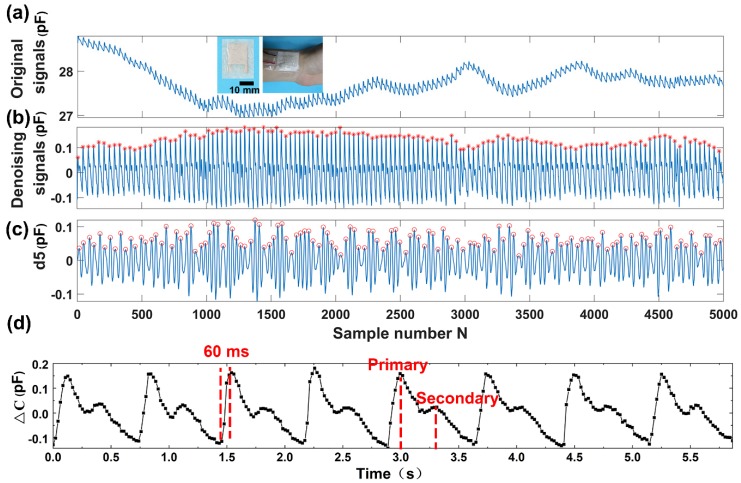
Detection and analysis of wrist pulse acquired by the capacitive sensor. (**a**) The recorded wrist pulse. (**b**) The wrist pulse after denoising and drift removal. (**c**) The 5th level wavelet high-frequency coefficient decomposed from the recorded wrist pulse signal (d5). (**d**) The enlarged denoising wrist pulse.

To evaluate the capacity of the napkin-based sensor for detection of the subtle physical signals, we apply the as-fabricated capacitive sensor to monitor wrist pulse. Pulse diagnosis, as a key diagnostic method in Traditional Chinese Medicine, has been employed in disease analysis for thousands of years. In modern medical practice, wrist pulse is also a key indicator of arterial blood pressure and heart rate [44,45]. It contains rich and critical information which can reflect the pathological changes of certain organs. The blood pulse wave is the external reflection of the heart and blood vessels’ important state information, and any change of our body system state will affect the pulse system. Thus, it provides a noninvasive and convenient way for effective diagnosis. To record the wrist pulse, the capacitive sensor is conformably attached to a tester’s wrist with PU tape, and it has a good response to the pulse beats. Figure 5a shows a pulse pressure signal acquired by the capacitive sensor. The collected signals contain baseline fluctuations due to motion artifacts and the respiration of the person. In order to remove that noise, baseline wanders removal by wavelet technique is done to minimize the distortion in the pulse signal. The envelope signals may include the low-frequency drift and high-frequency noise in maximum velocity. After wavelet decomposition of 5 levels using Daubechies wavelet transform db5, the collected wrist pulse wave is decomposed into low-frequency approximation coefficients (Figure S5) and high-frequency detail coefficients (Figure S6). By subtracting the 5th level wavelet approximation coefficients, the low-frequency drift of the waveform is eliminated (Figure 5b). The obtained denoising signals exhibit a periodically cyclic wave with regularly occurring systolic and diastolic waves. By analyzing the denoising signals (marked with stars) and the 5th level of high-frequency coefficients of the pulse wavelet transformation (marked with circles) (Figure 5c), we find that the pulse rate calculated from both signals are the same (70 beats per minute). The results indicate the 5th level high-frequency coefficients retain the main information of the blood pulse wave. In addition, it further verifies that the signals located in the baseline of the eye blinking wave discussed above are the blood pulse, because the same volunteer has unique pulse rate in the same situation. The subtle signals recorded during the eye blinking has the same cycle frequency as the blood pulse, which further proves that the sensor attached to the temple can not only distinguish eye blinking but also the blood pulse.

Moreover, the enlarged wrist pulse signals clearly exhibit the characteristic peaks of peripheral artery waveforms (Figure 5d). From the wrist pulse wave, we can obtain that the response time of the sensor is less than 60 ms, deduced from the rising time of the pulse. Under normal condition, a typical radial artery pulse waveform is obtained with two distinctively characteristic peaks [46]. The shape of the wave can be described as the superimposition of a primary wave and a secondary wave. As compared to the primary wave, the secondary wave has a relatively lower amplitude and a phase shift. The primary wave is generated when the left ventricle of the heart is in contraction forcing blood into the arterial structure, which usually contains information of the heart itself. The secondary wave is established by the wave reflection, which is an echo of the primary wave and usually occurring when the left ventricle of the heart is in diastole. The secondary wave contains information of the peripheral arterial system. The distinctive characteristic of wrist pulse caused by the rhythmic contraction and relaxation of the myocardium can be sensitively acquired by our capacitive sensor. The results demonstrate that our sensor has a great potential to serve as a wearable diagnostic device to monitor a human’s health in real-time.

## 4. Conclusions

In conclusion, we have developed a low-cost wearable napkin-based capacitive sensor, without the requirement of any expensive equipments and high skills. The napkin-based sensor prepared by the paper-based method is low cost, easy to fabricate, time-saving, eco-friendly, and has high sensitivity. Furthermore, the sensor is employed to detect finger motion, eye blinking, and even the subtle wrist pulse with detailed characteristic peaks in real time. Noteworthy, the capacitive sensors can detect multiple inputs simultaneously. Specifically, blood pulse wave can be registered at the same time while recording eye blinking and analyzed separately by wavelet transform analysis. These proof-of-concept demonstrations indicate that this sensor has a great potential for human–machine interface, prosthetics, and home-based healthcare.

## Supplementary Materials

The following are available online at https://www.mdpi.com/1424-8220/19/15/3427/s1, Figure S1: Sheet resistances of different densities of the silver NWs on the napkin, Figure S2: Optimization of the resolution of the patterned napkin tissue, Figure S3: Low-frequency approximation coefficients of the eye blinking signals, Figure S4: High-frequency detail coefficients of the eye blinking signals, Figure S5: Low-frequency approximation coefficients of the wrist pulse, Figure S6: High-frequency detail coefficients of the wrist pulse.
