# Supplementary material for: Low-Cost and Highly Sensitive Wearable Sensor Based on Napkin for Health Monitoring
Source: Sensors (Basel). 2019 Aug 5;19(15):3427. doi: 10.3390/s19153427 (PMC6695873; doi:10.3390/s19153427)
Supplement: Supplementary file 1 [file sensors-19-03427-s001.pdf]

sensors

Supplementary Materials: Low-Cost and Highly Sensitive Wearable Sensor Based on Napkin for Health Monitoring

Liping Xie [1,*], Peng Chen [2], Shuo Chen [1], Kun Yu [1], and Hongbin Sun [3]

1 College of Medicine and Biological Information Engineering, Engineering Research Center of Medical Imaging and Intelligent Analysis, Ministry of Education, Northeastern University, Shenyang 110169, China
2 School of Chemical and Biomedical Engineering, Innovative Centre for Flexible Devices, Nanyang Technological University, Singapore 637459, Singapore
3 Department of Chemistry, Northeastern University, Shenyang 110819, China
* Correspondence: xielp@bmie.neu.edu.cn

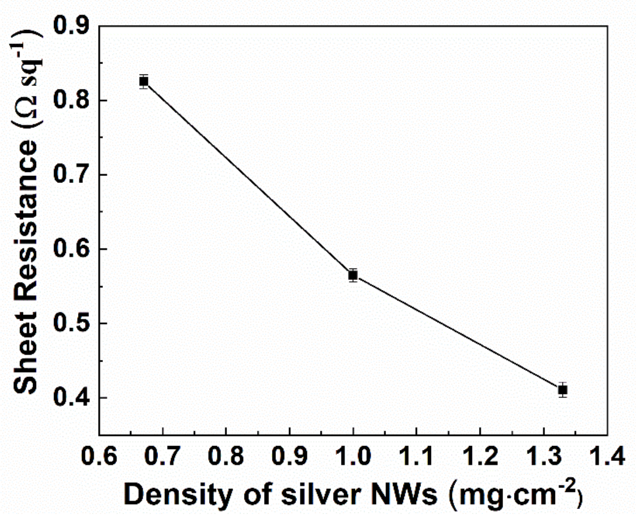

Figure S1. Sheet resistances of different densities of the silver NWs on the napkin.

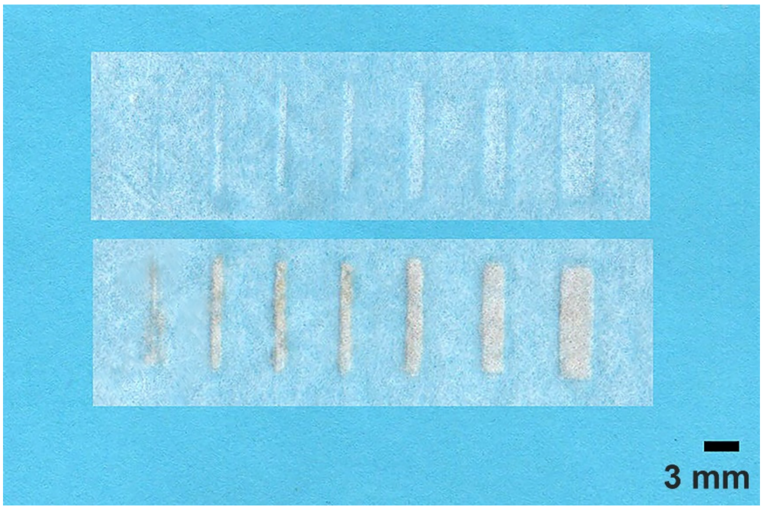

Figure S2. Optimization of the resolution of the patterned napkin tissue.

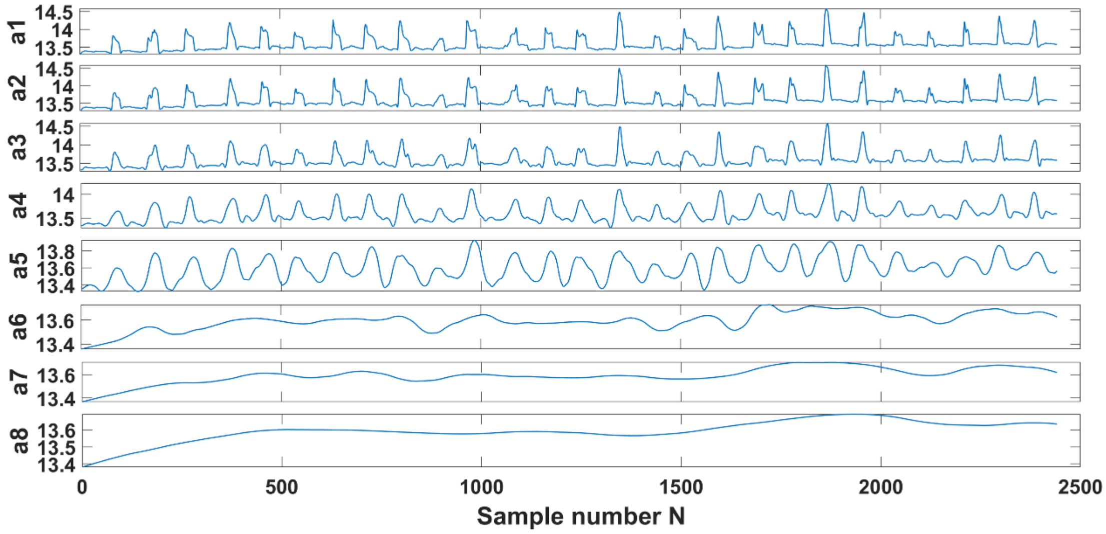

Figure S3. Low-frequency approximation coefficients of the eye blinking signals.

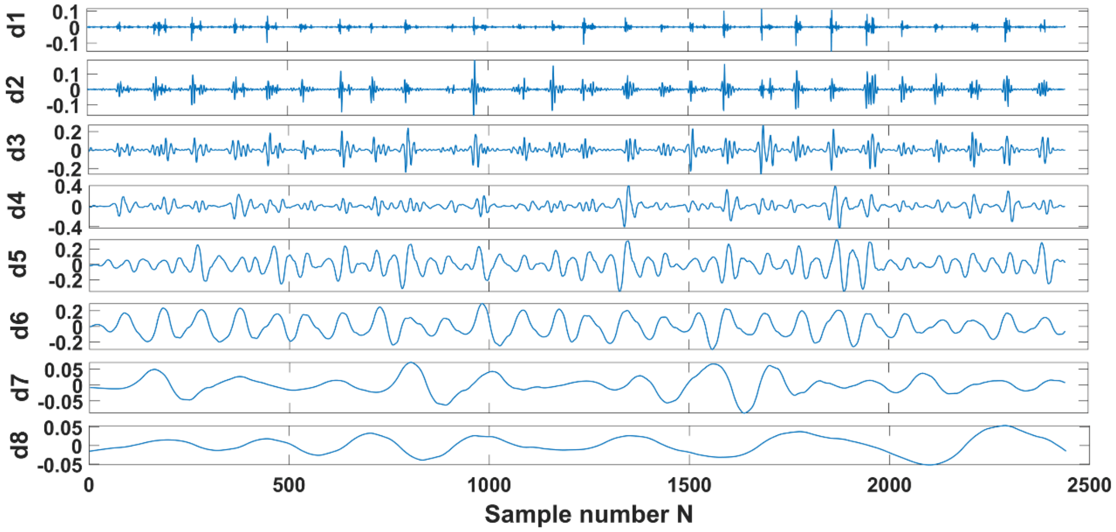

Figure S4. High-frequency detail coefficients of the eye blinking signals.

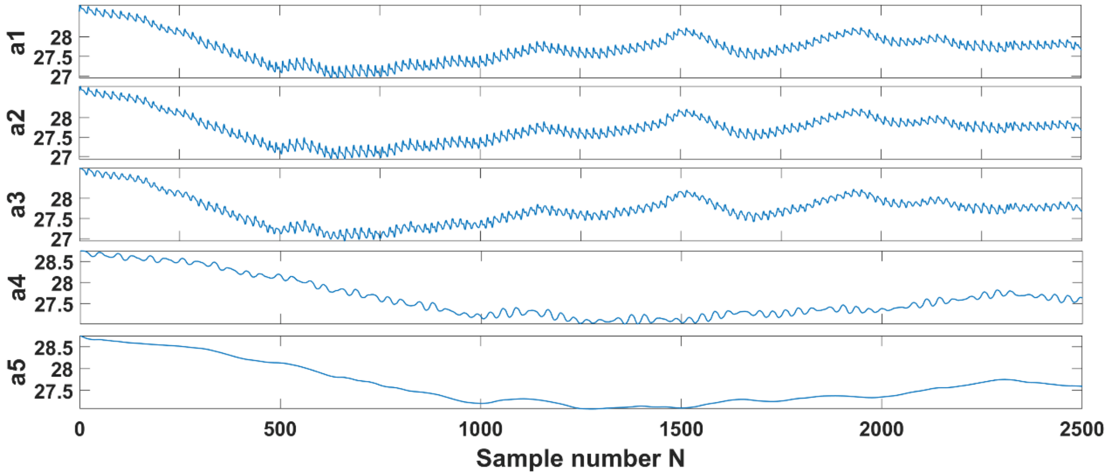

Figure S5. Low-frequency approximation coefficients of the wrist pulse.

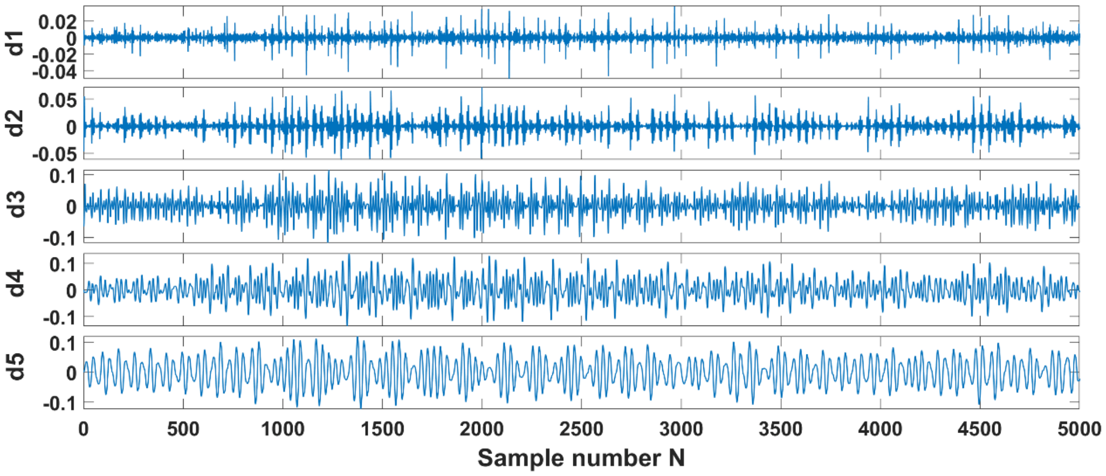

Figure S6. High-frequency detail coefficients of the wrist pulse.
